# Methane Adsorption on Aggregates of Fullerenes: Site-Selective Storage Capacities and Adsorption Energies

**DOI:** 10.1002/cssc.201300133

**Published:** 2013-06-06

**Authors:** Alexander Kaiser, Samuel Zöttl, Peter Bartl, Christian Leidlmair, Andreas Mauracher, Michael Probst, Stephan Denifl, Olof Echt, Paul Scheier

**Affiliations:** a Institut für Ionenphysik und Angewandte Physik, Universität InnsbruckTechniker Str. 25, 6020 Innsbruck (Austria); b Department of Physics, University of New HampshireDurham, NH 03824 (USA)

**Keywords:** adsorption, density functional calculations, mass spectrometry, molecular dynamics, nanoparticles

## Abstract

Methane adsorption on positively charged aggregates of C_60_ is investigated by both mass spectrometry and computer simulations. Calculated adsorption energies of 118–281 meV are in the optimal range for high-density storage of natural gas. Groove sites, dimple sites, and the first complete adsorption shells are identified experimentally and confirmed by molecular dynamics simulations, using a newly developed force field for methane–methane and fullerene–methane interaction. The effects of corrugation and curvature are discussed and compared with data for adsorption on graphite, graphene, and carbon nanotubes.

## Introduction

Adsorption of hydrogen, methane, and other hydrocarbons in porous carbonaceous materials shows promise for high-density storage of hydrogen-rich molecules that may one day be used to power light-duty vehicles.^1^–^5^ However, efficient on-board storage of hydrogen or methane presents a major technological challenge. For cars, some 10 kg of hydrogen need to be stored to achieve driving ranges greater than 300 miles (500 km). The 2017 target stated in the *2011 Interim Update of the Hydrogen Storage Engineering Center of Excellence* is to store 5.5 wt % of hydrogen at a volumetric density of 0.040 kg L^−1^, with an ultimate target of 7.5 wt % at 0.070 kg L^−1^.^5^ The most promising alternatives for on-board storage of hydrogen are storage in form of metal hydrides, chemical compounds, or physisorption on light-weight adsorbents with large surface areas.^4^ Storage of gaseous or liquid H_2_ in tanks at very high pressures and/or very low temperatures is not likely to meet the targets and raises major safety issues.

For hydrogen sorption, carbon is the material of choice because of its low weight and benign environmental properties. However, the adsorption energies on the surfaces of pristine graphene, nanotubes, and fullerenes are only 0.04–0.05 eV, well below the target of 0.1–0.4 eV (10–40 kJ mol^−1^) set by the Hydrogen-Sorption Center of Excellence funded by the US Department of Energy.^6^ Values below 0.1 eV would require cryogenic temperatures combined with high pressures, which would decrease system efficiency and increase system cost; values above 0.4 eV would require temperatures well above ambient for release of H_2_ and thus compromise energy efficiency and safety.

Several strategies exist that promise increased adsorption energies; most of them involve either impurities, intrinsic defects, or multi-wall interactions, for example for adsorption sites in the interior of narrow nanotubes, or in the grooves between parallel nanotubes. Early research had indeed raised hopes that single-walled nanotubes would be able to store hydrogen at room temperature at 6 wt % and even higher.^7^ However, the most promising results were found to be incorrect due to measurement errors and the presence of uncontrolled impurities, and no correlation could be established between the reported hydrogen capacities and various properties of the nanotube structures or synthesis methods.^8^

Thus, research involving nanotubes faces a fundamental dilemma: Defects are desirable because they increase adsorption energies; on the other hand, the non-uniformity of actual samples of nanotubes and the presence of unspecified defects makes it nearly impossible to determine the nature and energies of adsorption sites.^9^–^12^ Consequently, in 2006 the Department of Energy decided to discontinue applied research and development investment in pure, undoped single-walled carbon nanotubes for vehicular hydrogen storage applications.^8^ Until today, the synthesis of aligned, strictly uniform single-walled nanotubes poses a major challenge.^13^ Experiments on bundles of nanotubes suffer from non-uniform tube diameters, different tube chiralities, and defects including the presence of nanosized metal and metal-oxide particles that are used in the catalytic chemical vapor deposition (CCVD) technique, other impurities, and topological defects such as vacancies, non-hexagonal carbon rings, and the presence of uncapped tubes.

Experiments on C_60_ that can be synthesized at very high purity and free of defects offer a way out of this dilemma. Adsorption of hydrogen or methane on pristine or functionalized fullerenes has already been subject to several theoretical studies.^3^, ^14^–^22^ Our group computed a value of 49.5 meV for hydrogen adsorbed on pristine, isolated C_60_ by using the ωB97X-D functional without zero-point correction; the value decreased to 37.5 meV for the PBE0 functional. The values are in good agreement with a value of 32 meV obtained by using the symmetry-adapted perturbation theory,^21^ and 52 meV by using local spin density approximation including the counterpoise correction.^20^ The values agree closely with theoretical and experimental results obtained for H_2_ adsorbed on nanotubes^23^ and graphene.^24^

Higher adsorption energies have been computed for C_60_ exohedrally doped with one or more metal atoms.^3^, ^14^–^18^ Alkali and earth alkaline atoms are found to reside on top of hexagonal or pentagonal sites. The high electron affinity of C_60_ results in electron transfer from the metal to the fullerene, thus enhancing the binding of hydrogen to the metal atoms. The binding energies calculated for alkali or earth alkaline atoms to the fullerene exceed the cohesive energies of the bulk metals, thus avoiding undesirable clustering of metal atoms.^14^–^16^ Adsorption energies just below 0.1 eV per hydrogen molecule have been computed for sodium,^15^, ^18^ 0.17 eV for lithium,^18^ and 0.2–0.4 eV for strontium and calcium.^16^

However, experiments involving fullerenes are scarce. Saha and Deng reported that the hydrogen adsorption capacity of solid C_60_ at 77 K and 120 bar could be tripled to 13 wt % upon controlled oxidation of the sample although the adsorption isotherms indicated a heat of adsorption of only 25 meV.^25^ Yamada et al. investigated hydrogen adsorption on a C_60_ monolayer deposited on a Cu(111) surface by helium scattering.^26^ Thermal desorption of a hydrogen monolayer resulted in a desorption peak at 437 K from which the authors estimated a binding energy of 1.2 eV. Mauron et al. investigated adsorption of hydrogen in sodium-intercalated fullerenes (i.e., sodium fullerites).^27^ They concluded that chemically bound fulleranes including C_60_H_36_ are formed in the experiment. Teprovich et al. reported on reversible chemisorption of hydrogen with lithium-doped C_60_ to form fulleranes.^28^ The binding energies obtained in these latter studies^26^–^28^ greatly exceed the optimal range for sorbent materials.^6^

An alternative experimental approach involves individual, free C_60_ molecules rather than C_60_ solids. Metallofullerene complexes such as Ca_32_C_60_ have been synthesized in helium gas and identified in mass spectra,^29^ but adsorption of H_2_ or other gases on these complexes has not yet been investigated.

We have recently designed a new method to study adsorption of atoms and polar or nonpolar molecules on C_60_ or C_70_ by doping cold (0.37 K), superfluid helium nanodroplets^30^ with fullerene plus H_2_O, NH_3_, He, H_2_, CH_4_, or other molecules.^31^–^33^ Nonpolar species such as He, H_2_, CH_4_ show a propensity to form a commensurate layer where each carbon hexagon and pentagon adsorbs one particle although He and H_2_ are sufficiently small to allow for adsorption of additional particles into the first adsorption layer.

Here, we present a first detailed study of adsorption on free aggregates of fullerenes (some results were already reported in a recent Letter^33^). We chose the C_60_–CH_4_ system because our experimental results for this system are the most comprehensive. Although hydrogen is the primary candidate for fuel cells or internal combustion engines, CH_4_ is also of interest because of its low toxicity and dominance in natural gas.^1^, ^34^ Room-temperature storage on graphitic nanostructures appears more feasible for CH_4_ because the physisorption energies for CH_4_ are twice as large as for hydrogen; numerous experimental and theoretical investigations of CH_4_ and other small hydrocarbon molecules adsorbed on graphite,^35^–^39^ graphene,^40^ nanotubes,^9^, ^10^, ^12^, ^37^, ^39^, ^41^, ^42^ and layers of C_60_^19^ have been reported.

The experiment involves mass spectrometry, which allows us to determine the exact number of adsorption sites for fullerene aggregates containing up to five C_60_. The nature and adsorption energies of the sites in C_60_ aggregates are determined by density functional theory (DFT) and molecular dynamics (MD) simulations. The C_60_ aggregates offer a hierarchy of adsorption sites including sites in the grooves between pairs of adjacent fullerenes that are analogous to groove sites between two parallel single-walled nanotubes and dimple sites between triplets of C_60_, analogous to dimple sites that exist over hexagonal close-packed layers of C_60_.^19^ Calculations are presented for aggregates containing up to four C_60_ molecules. As the experimental results pertain to positively charged complexes we present calculations for neutral as well as charged systems; they display the expected^43^ increase of adsorption energies upon charging. Excellent agreement between experiment and theory is obtained for the adsorption capacity in these various sites. The adsorption energy increases from 118 meV over hexagonal sites to 220 meV for groove sites and 281 meV for dimple sites; these values are in the optimal range.^6^

## Results and Discussion

### Experimental

A mass spectrum of helium droplets doped with C_60_ and CH_4_ is displayed in Figure 01. The most prominent mass peaks are due to aggregates that contain up to five C_60_ but no CH_4_ molecules. The absence of fragments such as C_118_, C_116_ etc. that are characteristic dissociation products of highly excited fullerene dimers^44^ indicates that the ionized aggregates consist of intact C_60_ units. The partial pressure of CH_4_ was kept low to avoid spill-over of one series, such as C_60_–CH_4_, into the next series, (C_60_)_2_–CH_4_.

**Figure 1:**
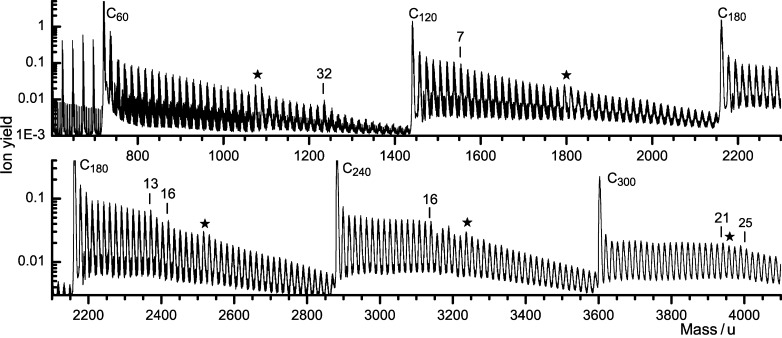
A mass spectrum of helium droplets doped with C_60_ and CH_4_, showing the adsorption of CH_4_ molecules at aggregates that contain as many as five C_60_ molecules. Some abrupt changes in the ion yield have been marked. Stars indicate contributions from doubly charged fullerene aggregates.

Figure 01 reveals several anomalies in the otherwise smooth ion yield of fullerene–methane complexes; labels above prominent anomalies indicate the number of methane molecules. Stars indicate mass peaks that are contaminated by contributions from doubly charged fullerene trimers, pentamers, and so on; these contaminations can be avoided by reducing the partial pressure of C_60_.

Each mass peak in Figure 01 actually consists of several closely spaced peaks that arise from i) intramolecular dissociation of methane, ii) ion–molecule reactions in the fullerene–methane complexes, and iii) contributions from isotopologues containing one or more ^13^C isotopes (natural abundance 1.07 %). The individual peaks are well resolved for methane adsorbed on fullerene monomers and dimers. We corrected the ion yield for the (substantial) contributions from ^13^C isotopes by using a matrix method as described in a recent publication^45^ and thus obtained the abundance of isotopically pure (^12^C only) ions.

**Figure 2:**
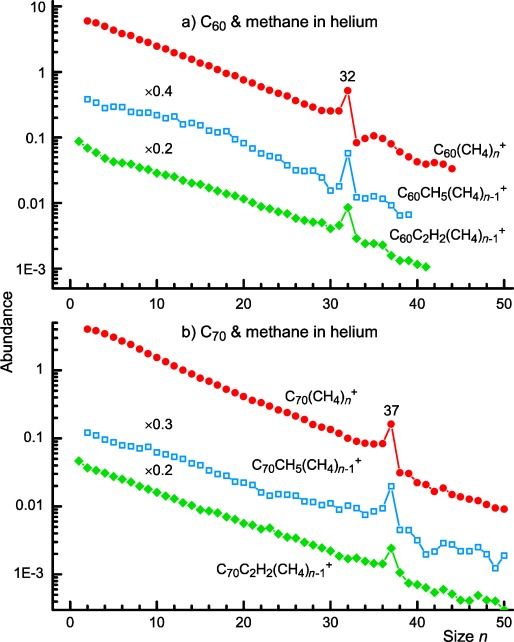
a) Ion abundance (corrected for contributions from species containing one or more ^13^C atoms) of the most prominent ion series in the mass spectra, namely C_60_M(CH_4_)_*n*−1_^+^ with M=CH_4_ (stoichiometric ions), M=CH_5_, and M=C_2_H_2_. b) As in (a) but for C_70_.

Figure 2 a displays the ion abundance of the three most intense ion series for the C_60_ monomer, namely C_60_(CH_4_)_*n*_^+^, C_60_(CH_4_)_*n*−1_CH_5_^+^, and C_60_(CH_4_)_*n*−1_C_2_H_2_^+^. All three series exhibit a strong local maximum at *n*=32. Similarly, Figure 2 b reveals that C_70_–methane complexes are particularly abundant when 37 molecular units are bound to the fullerene ion.

Anomalies in the ion abundances are revealed more clearly if the distribution is divided by a smooth function. Figure 03 summarizes the corresponding data for all stoichiometric ion series, (C_60_)_*m*_(CH_4_)^+^. The smooth functions were computed from local averages of the experimental ion abundance with Gaussian weighting.^46^ By definition, these relative ion abundances average to 1; local deviations from 1 reflect anomalies in the relative dissociation energies.^47^ In the special case that the heat capacities of the cluster ions are small compared to the classical equipartition value one finds that the relative ion abundances are directly proportional to the relative dissociation energies.^48^ The dissociation energy *D*_n_ of a complex C_60_(CH_4_)_*n*_^+^ is the energy of Reaction (1):
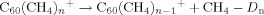
(1)

**Figure 3:**
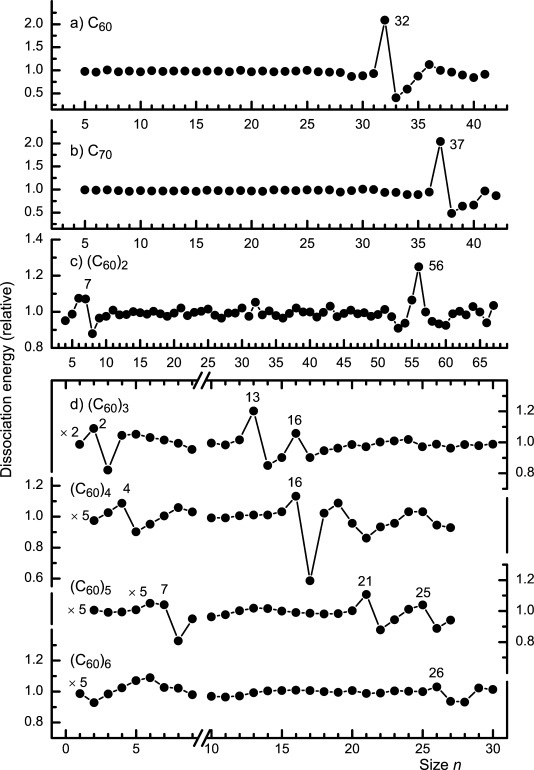
Ion abundance of stoichiometric ions C_*m*_(CH_4_)_*n*_^+^ after division by a smooth function. Anomalies in these relative abundances are expected to be approximately proportional to relative dissociation energies, that is, the energy needed to remove one CH_4_ molecule from the complex.

In other words, *D*_n_ is the energy required to evaporate the least-bound molecule from the complex. Thus the data in the upper panels of Figure 03 indicate that molecules in C_60_(CH_4_)_32_^+^ and C_70_(CH_4_)_37_^+^ are twice as strongly bound as the average; an additional methane molecule adsorbed on these tightly bound complexes is a factor two more weakly bound.

The dimer ion series (C_60_)_2_(CH_4_)_*n*_^+^ (Figure 3 c) shows anomalies at *n*=7 and 56. The nominal mass of (C_60_)_2_(CH_4_)_56_^+^ coincides with that of (C_60_)_3_(CH_4_)_11_^+^, but the actual masses differ by 1.41 u, so it is possible to resolve the ions. Furthermore, by adjusting the vapor pressures of C_60_ and CH_4_ in the pick-up cells, the abundance of the dimer ion series could be made much larger than that of the trimer ion series.

The relative dissociation energies for CH_4_ adsorbed on C_60_ trimer through hexamer ions are displayed in Figure 3 d. Local maxima in the relative dissociation energies that are at least 10 % above the average values occur at *n*=13 and 16 for the trimer, 16 for the tetramer, and 21 and 25 for the pentamer. An anomaly at *n*=26 for the hexamer is less significant.

Data for trimers and larger aggregates with fewer than ten adsorbed CH_4_ molecules are enhanced in Figure 3 d by factors 2 or 5; the dissociation energies exhibit local maxima at *n*=2 for the C_60_ trimer, 4 for the tetramer, and 7 for the pentamer. Even though the anomalies are weak they are statistically significant because the ion abundances are quite high.^49^

All observed anomalies are listed in Table 1. In the following section we will demonstrate that these data specify the maximum numbers of molecules in specific types of adsorption sites.

**Table 1:** Anomalies observed in the ion abundance together with features extracted from the computed energy and spatial distributions of methane molecules adsorbed on charged C_60_ aggregates.

C_60_ aggregate		Number of sites		
	experiment		theory	
		dimple	groove	full layer
monomer	32	–	–	32
dimer	7, 56	–	7	58
trimer	2, 13, 16	(2)	13	≈80
tetramer	4, 16	4	17	≈100
pentamer	7, 21, 25	–	–	–

### Computational

We have performed molecular dynamics (MD) simulations for the adsorption of 50, 80, or 500 methane molecules on neutral or positively charged aggregates containing up to four C_60_ molecules. Most simulations were run for 400 ps with time steps of 2 fs at a temperature of 4 K. The derivation of intermolecular forces between fullerenes, between methane and neutral or charged fullerenes, and between methane molecules, is discussed at the end of this paper.

Snapshots of singly charged fullerene aggregates with 80 CH_4_ molecules are shown in Figure 04; the snapshots were recorded at the end of simulation runs. CH_4_ molecules are depicted as tetrahedra to reveal their angular orientation. The color of the tetrahedra indicates their energy *E*, defined as the sum over all pairwise interactions with the fullerenes and all other CH_4_ molecules in the complex minus the sum of pairwise energies of the structurally unrelaxed aggregate with one missing CH_4_ molecule. According to this definition, the most strongly bound molecules in a complex have the lowest (most negative) values of *E*. We refer to −*E* as the binding energy.^50^ From Figure 04 one sees that the binding energies for the strongest-bound molecules increase as the number of C_60_ molecules in the complex increases (note that each panel in Figure 04 has a different energy-to-color conversion scale).

**Figure 4:**
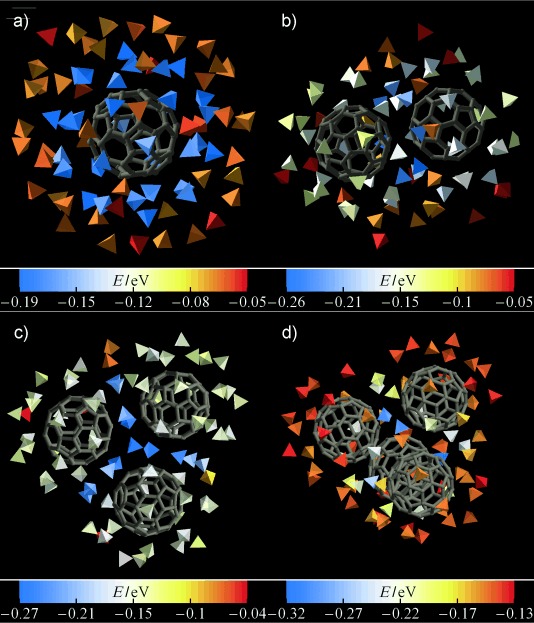
Energy-resolved snapshots of 80 methane molecules (shown as tetrahedra) adsorbed on charged fullerene aggregates. The monomer (a) is surrounded by a first shell of strongly bound (blue) methane molecules. The dimer (b) has the most strongly bound molecules in groove sites. For the trimer (c) and tetramer (d) dimple sites (blue) are most strongly bound, followed by other groove sites (white and yellow). The first adsorption layer is just saturated for the trimer but not yet for the tetramer.

The first solvation shell of C_60_^+^ is easily visible in Figure 4 a; the 32 most strongly bound molecules (colored blue) are adsorbed over the 12 pentagonal and 20 hexagonal faces. For the dimer (Figure 4 b), the most strongly bound (blue) CH_4_ molecules reside in the waist region; these adsorption sites will be referred to as groove sites. Other molecules in the first adsorption layer of the dimer are less strongly bound; they are colored white.

The snapshot of the trimer (Figure 4 c) reveals the enhanced binding in groove sites. Two of these groove sites offer particularly strong binding, namely the sites near the three-fold symmetry axis, so-called dimple sites (above and below the plane defined by the fullerenes). The C_60_ tetramer (Figure 4 d) has four such dimple sites.

Snapshots are not necessarily representative of the system over long times. To extract more reliable, quantitative data we have analyzed the geometry and energy of the systems over the last 50 ps of each simulation, with snapshots stored at increments of 1 ps. The histogram in Figure 05 displays the distribution of energies *E* for C_60_(CH_4_)_500_^+^. The 32 molecules that reside over the 20 hexagonal and 12 pentagonal faces form the narrow peak at *E*=−0.2 eV. The adsorption energies of the two sites differ by several percent; the sites will be characterized in more detail further below. The solid line in Figure 05 represents the cumulative sum of molecules with energies smaller than the indicated bin-energy *E*. The large energy gap in the histogram corresponds to the broad plateau in the cumulative sum. In the following we will merely show these cumulative sums, which allows for less congested Figures.

**Figure 5:**
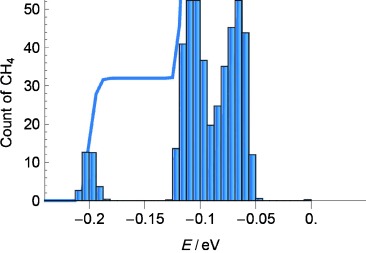
Histogram of the number of CH_4_ molecules with binding energies *E* for C_60_(CH_4_)_500_^+^. The solid line represents the cumulative sum of all molecules with energies smaller than *E*.

Figure 06 displays the energy distributions for the C_60_ monomer through tetramer for the most strongly bound CH_4_ molecules, where the energy of a CH_4_ molecule is defined as the sum over all pair-interactions with C_60_ and other CH_4_ molecules. The dimer features a distinct plateau at *n*=7, the trimer at *n*=13, and the tetramer at *n*=4 and 17. The distinctness of the plateaus depends slightly on the number of CH_4_ molecules in the simulation (either 50, 80, or 500). For C_60_ aggregates (excluding the monomer) with 500 CH_4_ molecules the plateaus become somewhat blurred because the contributions from the large number of molecules in outer layers are subject to thermal fluctuations. However, these simulations are valuable because they reveal additional plateaus at about 55–60 for the dimer, about 80 for the trimer, and about 100 for the tetramer.^51^

**Figure 6:**
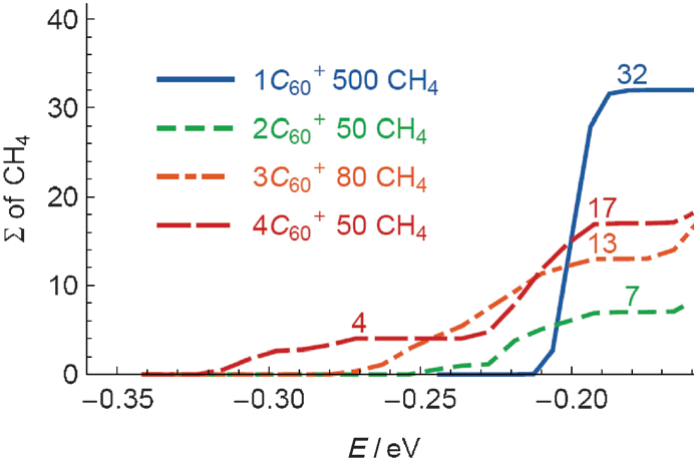
Counting the number of molecules in the adsorption layer of the charged C_60_ monomer through tetramer: The cumulative sums of molecules derived from the energy distributions (cf. Figure 05) reach plateaus when specific types of adsorption sites become saturated. One sees that there are four dimple sites for the tetramer; 7, 13, and 17 groove sites (including dimple sites) for the dimer through tetramer; and 32 face-centered sites for the monomer.

The energies of the four curves in Figure 06 cannot be compared directly to each other because of different occupations of the second and third solvation shells in our calculations. More insight was obtained by computing the energetics and geometry of a single CH_4_ molecule adsorbed on charged C_60_ aggregates; results are listed in Table 2. For C_60_^+^, hexagonal sites (117.8 meV) are preferred to pentagonal sites (107.9 meV) as already observed for H_2_ on C_60_.^45^ In its optimal configuration, CH_4_ resides 6.57 Å away from the center of the fullerene in the so-called “face” geometry. This structure was discussed for carbon nanotubes by Akai and Saito.^42^ They reported a maximum adsorption energy of 90 meV on the outside of the tube over a nanotube-hexagon and 96 meV on graphene. A comparative study of various dispersion corrected functionals applied to the methane–graphene system yielded adsorption energies in the range of 140–300 meV in the “face” geometry, denoted as “1d” by Thierfelder et al.^40^ Yang et al.^36^ studied CH_4_ adsorption on graphite with ab initio methods and reported 118 meV in the “face” configuration at low coverage, whereas Albesa et al.^38^ reported a heat of adsorption of 12.6 kJ mol^−1^ (131 meV) using Monte Carlo simulations. An experimental value of 11.3 kJ mol^−1^ (117 meV) was reported by Bienfait et al.^10^ for the isosteric heat of adsorption of CH_4_ on the external surface of a single-walled nanotube bundle. Our value of 117.8 meV agrees well with those data. As expected, adsorption energies decrease from flat to curved surfaces.

**Table 2:** Computational results for a single methane molecule adsorbed on a charged fullerene aggregate. min {*d*_i_} is the distance to the center of the nearest fullerene. Values were obtained by using the classical force field (as used in the MD simulation) and an optimization procedure.

C_60_ aggregate	Site	Energy [meV]	min{*d*_i_} [Å]
monomer	pentagon	107.9	6.70
monomer	hexagon	117.8	6.57
dimer	groove	218.4	6.57
trimer	dimple	280.9	6.71

The adsorption energy in a groove site of a fullerene dimer (218.4 meV) is 85 % larger than the energy in a single hexagonal site. The energy does not double because the perfect “face” arrangement of the hydrogen atoms over hexagons is not possible for groove sites. MP2 calculations of methane on carbon nanotubes predicted 126 meV for a single tube and 243 meV for a nanotube groove,^37^ an increase of 93 %. Absolute values are slightly larger compared to C_60_ due to the lower curvature. The adsorption energy increases to 280.9 meV for the (C_60_)_3_^+^ dimple site, 2.4 times the value over a hexagon of the C_60_ monomer ion. A geometry where one of the four vertices of methane points towards the center of all three fullerenes has been found; the minimal distance of the methane carbon atom to the center of one of the fullerenes increases from 6.57 for the monomer to 6.71 Å for the trimer.

The bare C_60_ dimer ion deserves some discussion. Its optimized (B3LYP/6-31g(d,p) without CP^52^ correction) structure is displayed in Figure 07. For a better view both spheres were cropped; only the two abutting hemispheres are shown. Both molecules are tilted, but the hexagonal faces stay nearly parallel; no lateral rotation was observed. A very similar structure was called the H–HH configuration and reported to have the highest binding energy for the neutral dimer.^53^ In this configuration a hexagon (H) is opposite a bond between two hexagons (HH). The calculated binding energy is −449 meV, stronger than −338 meV calculated by Zettergren et al.,^54^ but the equilibrium distance (10.24 Å) is the same. For comparison, an optimization by using the dispersion-corrected functional ωB97X-D yields a larger binding energy of −639 meV at a similar geometry and a smaller distance of 9.59 Å.

**Figure 7:**
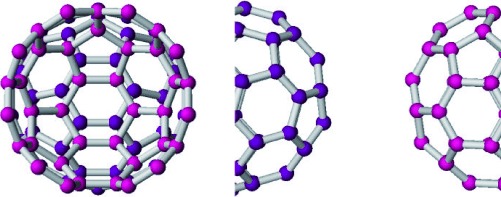
Part of the optimized fullerene dimer ion in front (axial) view (left) and side view (right). The fullerenes were cropped for the sake of clearer visualization.

What types of adsorption sites give rise to the plateaus in the energy distributions (Figure 06), that is, the gaps in the corresponding energy histograms? So far we inferred the nature of adsorption sites from individual snapshots. Statistically more accurate information is derived from spatial distributions that count molecules in specific adsorption sites. Let {*d*_i_} denote the set of distances of a specific methane molecule from the centers of all fullerenes in the aggregate, and min{*d*_i_} the distance from the nearest C_60_. For the monomer, min{*d*_i_} is simply the distance from the center of the fullerene; for the dimer min{*d*_i_} is the smaller of the two distances, etc. Gaps in a histogram plotted versus min{*d*_i_} will thus reveal geometrically distinct adsorption layers. The corresponding cumulative sums of these distributions, computed for 500 CH_4_ molecules adsorbed on C_60_ and its aggregates, are shown in Figure 08. All aggregates feature distinct plateaus between min{*d*_i_}=7.0 Å, where the first adsorption layer is completed, and 9.0 Å, where the second adsorption layer starts to build. The equilibrium distance of a single methane molecule adsorbed on C_60_^+^ ranges from 6.5 to 6.7 Å depending on the site and orientation of the molecule; the mean distance of the 32 nearest methane molecules in a simulation of C_60_(CH_4_)_50_^+^ is 6.69 Å.

**Figure 8:**
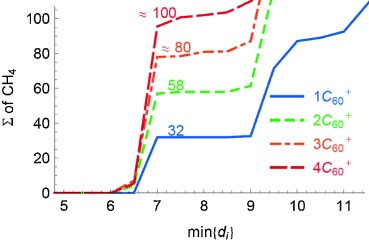
Cumulative sums of molecules derived from the spatial distributions reach plateaus when the first or, for the monomer, second adsorption layer are completed. min{*d*_i_} denotes the distance of a given CH_4_ molecule from the center of the nearest fullerene.

For the C_60_ dimer Figure 08 features a plateau at *n*=58 for the first adsorption layer, which agrees with the above-mentioned, less distinct plateau at about 55 to 60 in the energy distribution and the experimentally observed, distinct anomaly in the ion abundance at *n*=56. Unfortunately, it is not possible to experimentally test the predictions for the completion of the first adsorption layers at *n*≈80 and ≈100 for the C_60_ trimer and tetramer because of strong mass spectral interference with ions that contain additional C_60_ but fewer methane molecules.

The spatial distributions in Figure 08 do not reveal the number of groove or dimple sites because min{*d*_i_} has very nearly the same value for all molecules in the first adsorption layer. Instead, the following procedure is applied: As before, let {*d*_1_, *d*_2_, *d*_3_, …} denote the distances of a specific CH_4_ molecule to the centers of the fullerenes in the aggregate. Let *d*_i_ and *d*_j_ denote the two smallest values in this set. The molecule in question resides in a groove site if |*d*_i_−*d*_j_|<*ε*, where *ε* is a small, somewhat arbitrarily chosen distance that describes the approximate width of the groove region. For the dimer, the algorithm defines the region that encloses the plane bisecting the dimer axis. For the trimer and tetramer the regions defined as groove regions are illustrated in Figure 09, choosing *ε*=1 Å. Note that our definition does not limit the groove regions to the first adsorption layer, but the value of min{*d*_i_} helps to distinguish between groove sites that are part of the first adsorption layer and those that are further away.

**Figure 9:**
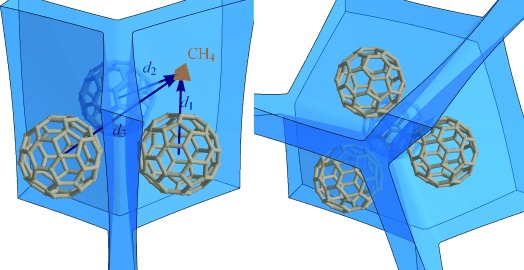
Groove sites for the a) trimer and b) tetramer ion, defined as the regions for which the distances to the two nearest C_60_ molecule are identical within 1 Å.

The calculated number of groove sites in the first adsorption layer is 7, 13, and 17 for the dimer through tetramer. The first two values are in perfect agreement with the experimental data (see Table 1); the value for the tetramer exceeds the experimental value by just one. The computed values are robust with respect to the choice of *ε* although *ε* has to be large enough to filter out small structural fluctuations.

The criterion for the definition of groove sites is easily extended to count the number of CH_4_ molecules in dimple sites (i.e., sites that have three nearest C_60_ molecules at approximately equal distances; they form a subset of groove sites). The results are, not surprisingly, *n*=2 for the trimer and 4 for the tetramer. The dimple sites of the tetramer are significantly more strongly bound than the remaining 13 groove sites; they form a plateau in the energy distribution, see Figure 06. On the other hand, the two dimple sites of the trimer do not form a distinct plateau in the energy distribution. This is probably due to contributions from the remaining molecules in (C_60_)_3_(CH_4_)_80_^+^ that are subject to thermal energy fluctuations.

**Figure 10:**
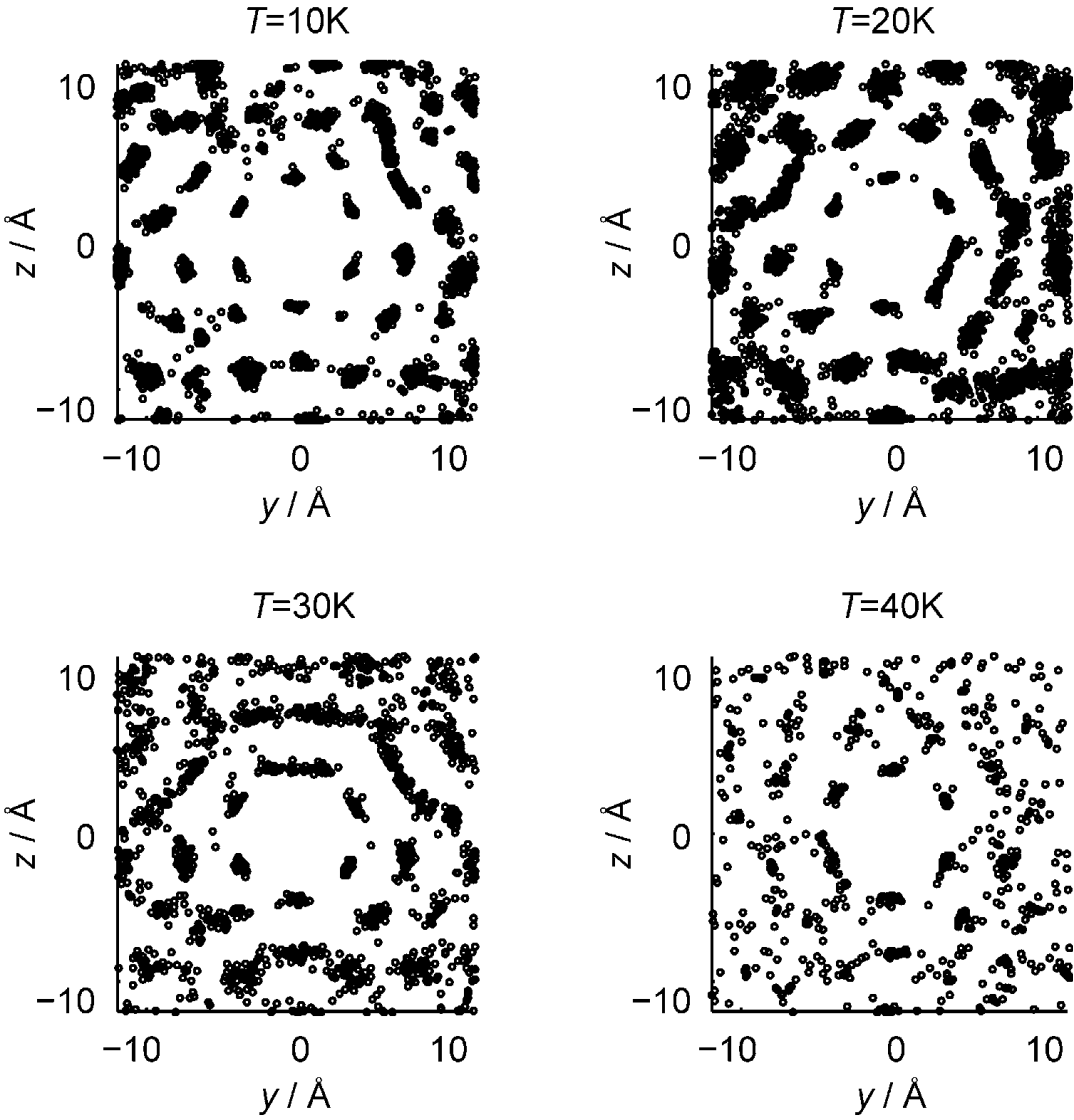
Location of CH_4_ molecules in groove sites of a fullerene dimer for different temperatures, showing a high degree of order at low temperatures. For each 1 ps time step of the simulation, the position of all molecules in the groove region is projected onto the plane that bisects the dimer axis.

Simulations discussed so far were performed at 4 K. The effect of temperature has been studied for the C_60_ dimer with 500 adsorbed CH_4_ molecules. Figure 10 shows the positions of all CH_4_ molecules that reside in the groove region, projected onto the plane that bisects the dimer axis. Projections were recorded every picosecond for the whole duration of a simulation. The pattern at 20 K is very regular. The CH_4_ molecules are arranged in a circular pattern and strongly localized, that is, there is radial and angular order, even far away from the innermost ring of seven molecules. At 30 K one still sees radial order in the first two or three rings, but at 40 K most of the order is lost except for the innermost ring.

**Figure 11:**
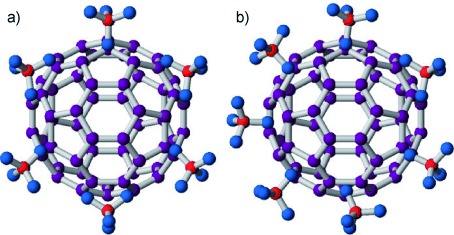
Methane molecules in groove sites of the C_60_ dimer ion with a) six or b) seven CH_4_ molecules. In this axial view the front fullerene is not shown to reveal the orientation of the adsorbate molecules more clearly.

At 10 K the position of the seven innermost methane molecules has changed. One of them (near the top in Figure 10) is displaced outwards; the remaining six form a nearly regular hexagon. This could perhaps signal that the packing of seven molecules in the groove is slightly too tight. To further investigate this, geometries were optimized with six, seven, and eight methane molecules in the groove. Starting at different temperatures we performed a step by step annealing in the MD runs and a final optimization run at 0 K. Starting at 30 K with six methane molecules, a highly symmetric structure is revealed that also reflects the symmetry of the substrate as shown in Figure 11 a. The orientation of the molecules alternates; either one (resembling a “goblet”) or two (resembling a “jumper” with hands up) hydrogen atoms are oriented inward. For a single molecule in the groove the jumper orientation is more favorable than the goblet configuration. The same was found for methane in grooves of nanotubes^12^.

For seven CH_4_ molecules the result depends on the initial temperature of the simulation. Starting at or below 20 K one obtains the result shown in Figure 11 b; all seven molecules reside in the groove with six in the goblet and one in the jumper orientation. If the simulation starts at 30 K, one of the seven molecules is pushed out of the groove to a nearby hexagon upon cooling, yielding a lower energy. The final arrangement resembles that in Figure 10 at 10 K. Simulations with eight CH_4_ molecules invariably result in one molecule being far outside the groove region. The average adsorption energies per molecule are 208, 194, and 184 meV for six, seven, and eight CH_4_ molecules, respectively.

Information about the structural order of the CH_4_ molecules in the grooves of the trimer may be inferred from their number, 13. The trimer has two dimple sites and three separate grooves. If the two dimple sites were occupied 11 molecules would have to reside in the three grooves, which does not allow for any regular arrangement. Instead, the molecules in the dimple sites might be displaced from the three-fold symmetry axis of the trimer, which will negatively impact on their binding energies. This may explain the absence of an energy gap in the calculated energy distribution, see Figure 06. Indeed, from a visual inspection of the MD trajectories we find that one of the dimple-site molecules is displaced from the exact dimple-site position.

For the tetramer, the calculated number of 17 molecules in groove sites including four in dimple sites would imply that 13 molecules reside in six grooves, again defying any regular arrangement. However, the experimental number is 16, which does allow for a highly symmetric arrangement, with four molecules in dimple sites plus two in each groove. The anomaly in the ion abundance and relative dissociation energy for this arrangement is accordingly large, see Figures 1 and 3 d.

## Conclusions

Intriguing new structures were identified by synthesizing fullerene–methane complexes within superfluid helium nanodroplets. These structures consist of methane molecules weakly adsorbed on charged fullerenes at moderate distances. Due to the high curvature of the fullerene a commensurate phase is possible, where each hexagon and pentagon of C_60_^+^ adsorbs exactly one molecule. For the C_60_ dimer, trimer, and tetramer ion the number of groove and dimple sites could be measured; the results agree closely with MD simulations. For the MD simulations a force field for fullerene–methane and methane–methane interactions was developed. Binding energies for adsorption on hexagons, pentagons, and in groove or dimple sites have been calculated and are in good agreement with corresponding data on nanotubes and other carbonaceous structures. A fullerene dimer supports six or seven molecules in the groove region at approximately 80 % higher binding energies than in the first adsorption shell of C_60_^+^; the trimer ion offers dimple sites that are 2.4 times as strongly bound.

## Experimental Section

Neutral helium nanodroplets were produced by expanding helium (purity 99.9999 %) from a stagnation pressure of 2 MPa through a 5 μm nozzle, cooled to about 8 K, into vacuum. The estimated average number of helium atoms per droplet formed in the expansion was of the order of 5×10^5^; the droplets were superfluid with a temperature of approximately 0.37 K.^30^ The resulting supersonic beam was skimmed by using a 0.8 mm conical skimmer, located 8 mm downstream from the nozzle. The skimmed beam traversed a 20 cm long differentially pumped pickup region containing methane (Linde 99.995 %) at partial pressures ranging from 1×10^−3^ to 4×10^−3^ Pa. A small amount of C_60_ (MER Corp., purity 99.9 %) or C_70_ (SES Research, 99 %) was vaporized into the pickup region from a crucible.

After the pickup region the doped helium droplets passed a region in which they were ionized by electron impact at 70 eV. Cations were accelerated to 40 eV into the extraction region of a commercial time-of-flight mass spectrometer equipped with a reflectron (Tofwerk AG, model HTOF); its mass resolution was about Δ*m*/*m*=1:5000. The base pressure in the mass spectrometer was 10^−5^ Pa. The ions were extracted at 90° into the field-free region of the spectrometer by using a pulsed extraction voltage. At the end of the field-free region they entered a two-stage reflectron, which reflected them towards a microchannel plate detector operated in single-ion-counting mode. Further experimental details have been described elsewhere.^32^, ^55^

### Computational methods

MD simulations were performed at a temperature of 4.0 K within the NVT ensemble using a Nosé–Hoover^56^ thermostat. Quantum chemically [B3LYP/6-31g(d,p)] optimized geometries of fullerene mono-, di-, tri-, and tetramers were space-fixed in the simulation. The fullerenes were surrounded by 50, 80, or 500 randomly distributed methane molecules. The initial condition is best described as a sparse cloud of methane molecules surrounding the fullerenes.

We used periodic boundary conditions with a large box size (1000 Å) to simulate vacuum conditions. In all simulations one of the fullerenes carried a charge of +1, distributed evenly on the C atoms. The methane molecules were treated as rigid bodies. The system was given enough time (400 ps) to obtain a stable configuration. We operated with an integration time step of 2 fs and stored snapshots of the trajectory every picosecond. A cutoff of 30 Å ensured a correct treatment of the long-range effects.

A new force-field for the C_60_–CH_4_, C_60_^+^–CH_4_, as well as for the CH_4_–CH_4_ interactions was developed. The C_60_^0,+^–CH_4_ energy surfaces of the neutral and charge species were sampled with DFT calculations by using the dispersion-corrected ωB97X-D functional.^57^ This functional has been shown to be a suitable tool to describe weak interactions such as hydrogen bonds and induced polarization with an accuracy comparable to MP2 and has proven its efficiency for various molecular systems.^58^ To sample the C_60_^0,+^–CH_4_ potential surface, five sites have been chosen: The centers of a hexagonal face, a pentagonal face, the bond between two hexagons, the bond between a hexagon and a pentagon, and the position of a C atom. One CH_4_ molecule was placed over these sites in three different rotational orientations and its distance *d* from the fullerene center was varied from *d*=6.5 to 15.5 Å; in total, 915 pair energies were calculated. The interaction was assumed to be zero at *d*=30 Å, and the total energies where shifted by the respective dissociation energies. It turned out that the potential energy depended considerably on the orientation of the CH_4_ molecule (by about 50 meV), thus prohibiting to approximate CH_4_ single sites (“united atom”) in the energy expression. In a fully relaxed optimization of the neutral C_60_–CH_4_ system the deformation energy of CH_4_ was only 1.8 meV, the total energy differed by only 0.2 eV, and the distance of the CH_4_ C atom to the center of C_60_ changed by −0.03 Å only while simultaneously tilting slightly to one side. The adsorption energy agreed within 2 % with the value of the non- relaxed system.

The CH_4_–CH_4_ potential surface was sampled in a similar procedure using coupled cluster calculations with single and double substitutions (CCSD^59^) in combination with the cc-pVTZ basis set.^60^ The basis set superposition error was accounted for by the counterpoise method. A total number of 816 points of the potential energy surface were calculated by scanning distances between 3 and 13 Å in 16 configurations. The CH_4_–CH_4_ interaction also depended strongly on the orientation of the molecules to each other. The CH_4_–CH_4_ system is ideally suited for benchmarking the ωB97X-D functional by comparison to our CCSD results. We found that ωB97X-D yielded relatively good adsorption distances within 5 % of the CCSD results. The trends in the adsorption energies were conserved with the ωB97X-D functional, but the absolute values were overestimated by a factor of two.

C_60_^0,+^–CH_4_ and CH_4_–CH_4_ force fields were then obtained by fitting the parameters of atom–atom pair potentials to the potential energy values. The functions and corresponding parameters are collected in Table 3. A thorough evaluation of the force field and the fitting will be given elsewhere.

**Table 3:** Pair potential energy functions, *V*(*r*), and values of the empirical coefficients *C*_12_ (in eV Å^12^), *C*_10_ (in eV Å^10^), *C*_6_ (in eV Å^6^), *A* (in eV), and *ρ* (in Å).

System	Interaction	Pair potential		Values of the parameters				
C_60_^+^–CH_4_	C–C	*C*_12_/*r*^12^-*C*_6_/*r*^6^	*C*_12_	60 316.2	*C*_6_	56.0586		
C_60_^+^–CH_4_	C–H	*C*_12_/*r*^12^-*C*_6_/*r*^6^	*C*_12_	1508.7	*C*_6_	0.86022		
C_60_–CH_4_	C–C	*C*_10_/*r*^10^-*C*_6_/*r*^6^	*C*_10_	4178.45	*C*_6_	52.1998		
C_60_–CH_4_	C–H	*C*_10_/*r*^10^-*C*_6_/*r*^6^	*C*_10_	301.922	*C*_6_	2.104		
CH_4_–CH_4_	C–C	*C*_10_/*r*^10^-*C*_6_/*r*^6^	*C*_10_	3.021	*C*_6_	22.2		
CH_4_–CH_4_	C–H	*A* e^−*r*/*ρ*^-*C*_6_/*r*^6^	*A*	21.34	*ρ*	0.4071	*C*_6_	8.411
CH_4_–CH_4_	H–H	*A* e^−*r*/*ρ*^-*C*_6_/*r*^6^	*A*	6.708	*ρ*	0.3603	*C*_6_	0.3642

The C_60_–C_60_ interactions were not treated with a force field during the MD-simulations. The fullerenes were kept fixed at optimized geometries [B3LYP/6-31G(d,p)] for the mono-, di-, trimer, and for the tetramer an additional fullerene was inserted manually to match tetrahedral symmetry at the average C_60_–C_60_ distance of the trimer. We did not expect significant improvements upon using relaxed fullerenes because of the large binding energy of the C_60_ dimer compared to C_60_–CH_4_.

All calculations were performed with the 6-31g(d,p) basis set,^52^, ^61^ which includes polarization functions.^62^ The Gaussian 09 A.02 program package^63^ was used for the quantum chemical calculations and the MD simulations were performed with the DL_POLY^64^ software.

## Notes

As a service to our authors and readers, this journal provides supporting information supplied by the authors. Such materials are peer reviewed and may be re-organized for online delivery, but are not copy-edited or typeset. Technical support issues arising from supporting information (other than missing files) should be addressed to the authors.
