# Supplementary material for: Methane Adsorption on Aggregates of Fullerenes: Site-Selective Storage Capacities and Adsorption Energies
Source: ChemSusChem. 2013 Jun 6;6(7):1235–44. doi: 10.1002/cssc.201300133 (PMC3799018; doi:10.1002/cssc.201300133)
Supplement: Supplementary file 1 [file cssc0006-1235-sd1.pdf]

CHEMISTRY & SUSTAINABILITY

CHEMSUSCHEM

ENERGY & MATERIALS

Supporting Information

© Copyright Wiley-VCH Verlag GmbH & Co. KGaA, 69451 Weinheim, 2013

Methane Adsorption on Aggregates of Fullerenes: Site-Selective Storage Capacities and Adsorption Energies

Alexander Kaiser,[a] Samuel Zöttl,[a] Peter Bartl,[a] Christian Leidlmair,[a] Andreas Mauracher,[a] Michael Probst,[a] Stephan Denifl,[a] Olof Echt,*[a, b] and Paul Scheier*[a]

cssc_201300133_sm_miscellaneous_information.pdf

Complete References (10 or more authors):

[11] A. Nikitin, X. L. Li, Z. Y. Zhang, H. Ogasawara, H. J. Dai, A. Nilsson, *Nano Lett.* **2008**, *8*, 162-167; M. Arai, S. Utsumi, M. Kanamaru, K. Urita, T. Fujimori, N. Yoshizawa, D. Noguchi, K. Nishiyama, Y. Hattori, F. Okino, T. Ohba, H. Tanaka, H. Kanoh, K. Kaneko, *Nano Lett.* **2009**, *9*, 3694-3698

[27] P. Mauron, A. Remhof, A. Bliersbach, A. Borgschulte, A. Zuttel, D. Sheptyakov, M. Gaboardi, M. Choucair, D. Pontiroli, M. Aramini, A. Gorreri, M. Ricco, *Int. J. Hydrogen Energy* **2012**, *37*, 14307-14314

[31] S. Denifl, F. Zappa, I. Mähr, F. Ferreira da Silva, A. Aleem, A. Mauracher, M. Probst, J. Urban, P. Mach, A. Bacher, O. Echt, T. D. Märk, P. Scheier, *Angew. Chem. (Int. Ed.)* **2009**, *48*, 8940-8943; S. Denifl, F. Zappa, I. Mähr, A. Mauracher, M. Probst, J. Urban, P. Mach, A. Bacher, D. K. Bohme, O. Echt, T. D. Märk, P. Scheier, *J. Chem. Phys.* **2010**, *132*, 234307; H. Schöbel, C. Leidlmair, P. Bartl, A. Aleem, M. Hager, O. Echt, T. D. Märk, P. Scheier, *Phys.Chem.Chem.Phys.* **2011**, *13*, 1092 - 1098; C. Leidlmair, Y. Wang, P. Bartl, H. Schöbel, S. Denifl, M. Probst, M. Alcamí, F. Martín, H. Zettergren, K. Hansen, O. Echt, P. Scheier, *Phys. Rev. Lett.* **2012**, *108*, 076101.

[63] M. J. Frisch, G. W. Trucks, H. B. Schlegel, G. E. Scuseria, M. A. Robb, J. R. Cheeseman, G. Scalmani, V. Barone, B. Mennucci, G. A. Petersson, H. Nakatsuji, M. Caricato, X. Li, H. P. Hratchian, A. F. Izmaylov, J. Bloino, G. Zheng, J. L. Sonnenberg, M. Hada, M. Ehara, K. Toyota, a. others, *Gaussian 09, Revision A.02*, Gaussian, Inc., Wallingford CT, 2009.
